# The Emerging Role of the FGF/FGFR Pathway in Gastrointestinal Stromal Tumor

**DOI:** 10.3390/ijms21093313

**Published:** 2020-05-07

**Authors:** Annalisa Astolfi, Maria Abbondanza Pantaleo, Valentina Indio, Milena Urbini, Margherita Nannini

**Affiliations:** 1 Department of Morphology, Surgery and Experimental Medicine, University of Ferrara, 44121 Ferrara, Italy; annalisa.astolfi@unife.it; 2 Department of Experimental, Diagnostic and Specialty Medicine, University of Bologna, 40138 Bologna, Italy; 3 “Giorgio Prodi” Cancer Research Center, University of Bologna, 40138 Bologna, Italy; valentina.indio2@unibo.it; 4 Biosciences Laboratory, Istituto Scientifico Romagnolo per lo Studio e la Cura dei Tumori (IRST) IRCCS, 47014 Meldola, Italy; milena.urbini@irst.emr.it; 5 Medical Oncology Unit, S.Orsola-Malpighi University Hospital, 40138 Bologna, Italy; margherita.nannini@aosp.bo.it

**Keywords:** gastrointestinal stromal tumor, GIST, FGF4, FGFR1, KIT, PDGFRA, SDH

## Abstract

Gastrointestinal stromal tumors (GIST) are rare neoplasms of mesenchymal origin arising in the gastrointestinal tract. The vast majority are characterized by mutually exclusive activating mutations in KIT or Platelet-derived growth factor alpha (PDGFRA) receptors, or less frequently by succinate dehydrogenase complex (SDH) or NF1 inactivation, with very rare cases harboring mutant BRAF or RAS alleles. Approximately 5% of GISTs lack any of such mutations and are called *quadruple* wild-type (WT) GISTs. Recently, deregulated Fibroblast Growth Factor (FGF)/FGF-receptor (FGFR) signaling emerged as a relevant pathway driving oncogenic activity in different molecular subgroups of GISTs. This review summarizes all the current evidences supporting the key role of the FGF/FGFR pathway activation in GISTs, whereby either activating mutations, oncogenic gene fusions, or autocrine/paracrine signaling have been detected in *quadruple* WT, SDH-*deficient*, or KIT-mutant GISTs.

## 1. Introduction

Gastrointestinal stromal tumors (GIST) are the most common mesenchymal tumors of the GI tract, arising from the interstitial cells of Cajal (ICCs) or their precursors. Since the identification of v-kit Hardy–Zuckerman 4 feline sarcoma viral oncogene homolog (KIT) or platelet-derived growth factor alpha (PDGFRA) activating mutations, GISTs have been considered one of the first examples of oncogene-addicted solid tumor, soon becoming the paradigm of precision oncology and leading to tyrosine-kinase inhibitors (TKIs) as the standard of care for this chemo-resistant rare tumor. Indeed, imatinib first, then sunitinib and regorafenib are recognized as the approved sequence of treatment for advanced GISTs and new TKIs, such as avapritinib and ripretinib, and are already in development [1,2,3,4,5]. As widely known, GISTs are extremely heterogeneous in clinical presentation and behavior, as well as in the molecular background underlying this heterogeneity and in the response to TKIs’ treatment. Although the vast majority of GISTs harbors KIT mutations that confer the highest sensitivity to TKIs, or more rarely PDGFRA mutations, about 10–15% of adult cases do not harbor any of these driver mutations and have historically been called as wild-type (WT). Among them, from 20% to 40% show loss of function of the succinate dehydrogenase complex (SDH), therefore defined as SDH-*deficient* GISTs [6,7,8]. This subgroup is readily identifiable on the basis of clinical features [6,9], of the global genomic hypermethylation driven by SDH loss [10], and of the specific gene expression profile, characterized by IGF1R overexpression and by the peculiar commitment to the neural lineage cell fate [11,12,13]. Conversely, about 15% of KIT/PDGFRA WT cases harbor mutations in BRAF/RAS or NF1 and are referred to as RAS-pathway (RAS-P) mutant GISTs [7,14,15,16]. The remaining cases, accounting for about 5% of all GISTs, are usually referred to as KIT/PDGFRA/SDH/RAS-P WT or *quadruple* WT (qWT) GISTs [17]. Notably, this specific subgroup shares a distinct transcriptome profile that is profoundly different from other GIST subtypes [18]. In particular, the qWT GISTs share the overexpression of NTRK2 and ETS-transcription factor family (ERG), that may play a potential role in their pathogenesis [18]. However, to date a consensus on the recurrent oncogenic driver has still not been found, whereas many different and often private mutational events have been reported, confirming the great molecular heterogeneity of this subgroup of GISTs [19].

Indeed, an ETV6–NTRK3 gene fusion was the first rearrangement to be described [20,21]. Moreover, two fusion genes involving FGFR1 (FGFR1–HOOK3 and FGFR1–TACC1) and other chimeric fusions (KIT–PDGFRA, MARK2–PPFIA1 and SPRED2–NELFCD) have been reported [7,21,22]. Finally, relevant somatic mutations, including TP53, MEN1, MAX, CHD4, FGFR1, CTDNN2, CBL, ARID1A, BCOR, and APC were also identified [7,21,22,23].

The fibroblast growth factor signaling pathway relies on a family of receptor tyrosine kinases (FGFR) and eighteen extracellular ligands (FGFs) and has been implicated in the oncogenic process of different tumor histotypes. It regulates many physiological processes, in both the embryonic and adult stages of development, such as tissue differentiation and homeostasis, angiogenesis, and wound healing [24].

In this scenario, there is a growing interest on FGFR pathway deregulation in GISTs, since FGFR fusion events, together with FGFR mutations and FGFR ligand overexpression represent the most frequent molecular alterations identified in KIT/PDGFRA WT GISTs so far, suggesting its likely pathogenetic role and providing a rationale for targeted therapeutic approaches [21,22,25,26]. Moreover, some evidences on FGFR events and imatinib resistance in KIT/PDGFRA mutant GISTs have been described [27,28,29,30,31,32].

The aim of this review is to report all current data about the FGFR pathway deregulation in GISTs, focusing on the current clinical implications and future perspectives.

## 2. FGF/FGFR Family

The human FGFR family consists of four members: FGFR1–4, which are transmembrane receptors with tyrosine kinase activity belonging to the immunoglobulin (Ig) superfamily that can be stimulated and activated by extracellular ligands. FGFR family members display an amino acid sequence highly conserved between members and throughout evolution and differ in their ligand binding capacity and tissue-specific distribution [33]. A fifth family member, lacking the tyrosine kinase domain, FGFR5 or FGFRL1, was discovered on the basis of interaction with FGFR-binding ligands [34]. FGFRL1 is thought to act as a decoy receptor negatively regulating FGFR signaling, therefore inhibiting cellular proliferation and inducing differentiation [34].

**Figure 1:**
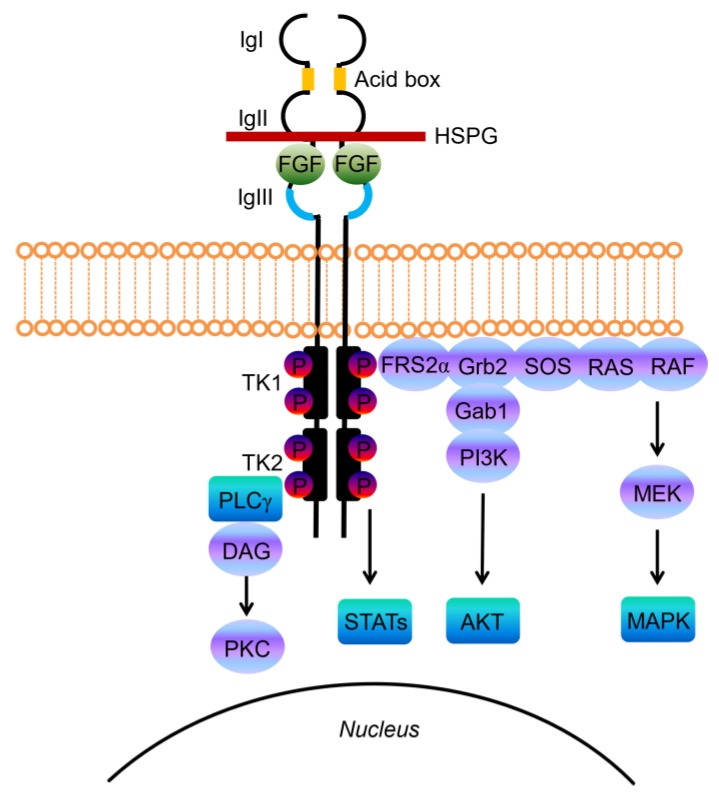
Fibroblast Growth Factor Receptor (FGFR) structure, ligand binding and signaling. Schematic representations of FGF receptor tyrosine kinase structure, composed of several domains including three extracellular immunoglobulin-like domains (IgI, IgII, and IgIII) and the acid-box, a transmembrane domain, and two intracellular tyrosine kinase domains (TK1 and TK2). The basic structure of the FGF/FGFR complex includes two receptor molecules, two FGFs, and one heparan sulphate proteoglycan (HSPG) chain. The signal transduction network activates four key downstream pathways: RAS/RAF/MAPK, PI3K/AKT, STATs and PLCγ.

Structurally, FGF-receptors are composed of a large extracellular ligand-binding domain, a single transmembrane helix and an intracellular portion containing two split tyrosine kinase domains (Figure 1) [33]. The extracellular portion contains three immunoglobulin-like (Ig-like) domains, with a linker region between the first and second Ig-loop containing a highly conserved stretch of glutamate-, aspartate-, and serine-rich sequence, called the acid-box **(**Figure 1) [35]. The second and third Ig-domains are involved in FGF binding and regulate ligand-binding specificity, while the first one and the acid-box mediate receptor auto-inhibition [33,35].

The specificity of ligand binding in FGFR1–3 is mainly determined by alternative splicing of the third Ig-domain, producing three possible IgIII isoforms. While IgIIIa is encoded by exon 7 alone, IgIIIb and IgIIIc derive from alternative splicing of the invariant exon 7 and one of two mutually exclusive exons, either exon 8 or exon 9, respectively [36,37,38]. Alternative splicing does not occur in FGFR4 that is expressed as a single isoform paralogous to the FGFR-IIIc isoform, due to the absence of an alternative exon [39]. Since the Ig-loop III is in the core portion of the ligand-binding site, alternative splicing profoundly alters the ligand spectrum of the receptor, with the IIIc variant being able to bind a wider number of FGF ligands [40]. Different ligand-binding specificity is also linked to the different tissue distribution of the receptors, with epithelial tissues expressing predominantly the IIIb isoform and mesenchymal tissues expressing the IIIc [40].

FGFs, the native ligands of FGF receptors, belong to a family that includes 22 members identified by sequence homology, which were grouped into subfamilies based on function or phylogenetic relation. Eighteen of these ligands encode molecules known to signal through FGF tyrosine kinase receptors. The canonical secreted FGFs can belong to the Fgf1, Fgf4, Fgf7, Fgf8 and Fgf9 subfamily, and exert the prototypical FGF functions that control cell proliferation, differentiation and survival, by binding and activating FGFRs [39]. The Fgf15/19 subfamily members have evolved to function as endocrine factors, regulating phosphate, bile acid, carbohydrate, and lipid metabolism [41]. The Fgf11 subfamily of genes encode intracellular FGFs, which are non-signaling proteins. Canonical FGFs are tightly bound to heparin/heparan sulfate (HS) proteoglycans (HSPGs), which function to limit diffusion through the extracellular matrix (ECM), but also serve as cofactors since cell surface HPSGs stabilizes the FGF ligand–receptor interaction, forming a ternary complex with FGFR that enhances receptor binding and signaling [42]. The endocrine FGFs evolved with reduced affinity for heparin/HS, and as a consequence, they are able to diffuse away from their cells of origin and enter the circulation, but require members of the Klotho family of proteins (αKlotho, βKlotho, or KLPH) for high affinity receptor binding [43].

Upon ligand binding the receptor undergoes dimerization, which leads to a conformational shift in receptor structure that subsequently induces a trans-autophosphorylation event of the intracellular kinase domain, driving the activation of downstream signal transduction pathways (Figure 1) [41]. The phosphorylated tyrosines are recognized and bound by adaptor proteins that contain Src homology 2 (SH2) domains or phospho-tyrosine binding (PTB) domains. FGFR substrate 2 (FRS2) is an adaptor protein largely specific to FGFR that, upon phosphorylation by activated FGFR, recruits other adaptor proteins (SOS, GRB2) that end up in activating RAS and the downstream RAF/MAPK pathway, mainly involved in mediating cellular proliferation [39,44]. Alternatively, GRB2 can also bind GAB1 and activate the PI3K/AKT axis, promoting cell survival. Lastly, direct phosphorylation by the activated FGFR tyrosine kinase stimulates other pathways, such as the signal transducers and activator of transcription (STAT) pathway, or phospholipase Cγ (PLCγ). The latter leads to the production of phosphatidylinositol-3,4,5-triphosphate (PIP3) and diacylglycerol (DAG), and activates the downstream protein kinase C (PKC) signaling [39,41,44].

Deregulation of FGF signaling can be caused by mutations that drive ligand-independent receptor signaling or alterations that support ligand-dependent receptor activation, and is responsible for tumor development and progression or resistance to therapies across many tumor types [24,45,46].

Enhanced FGF signaling in tumors is mediated by either receptor amplification, gene fusion or mutation, or by ligand-dependent activation, such as autocrine or paracrine signaling. For example FGFR1 amplification is recurrently seen in less than 20% of squamous non-small-cell lung carcinoma (NSCLC) and breast cancer, while somatic activating mutations are more common in FGFR2 and FGFR3, with FGFR2 mutations involving 10–12% of endometrial carcinomas and approximately 4% of NSCLCs and gastric cancers, and FGFR3 being highly recurrent in urothelial carcinomas [24,46,47]. Moreover, oncogenic gene fusions have been recurrently discovered in a number of cancers. For example, FGFR3 fusions are relatively common in glioblastoma and bladder cancer, while FGFR2 chimeric transcripts are frequent in lung squamous cell carcinomas and intrahepatic cholangiocarcinomas [46,47]. Several studies have also suggested that aberrant FGF and/or FGFR signaling has pleiotropic effects on tumor cells and the surrounding stroma, that include proliferation induction, resistance to cell death, increased motility and invasiveness, enhanced metastasis, and resistance to chemotherapy [46,47].

## 3. FGF/FGFR Events in GIST

The contribution of FGF/FGFR signaling to GIST pathogenesis was established in different molecular subgroups of this disease with various levels of evidence and involvement. For example, the extensive molecular characterization of *quadruple* WT GISTs in search of the causal oncogenic event was the driving force for the identification of activating alterations in FGF receptors, including point mutations and gene fusions, and of FGFR ligand overexpression (Table 1).

**Figure 2:**
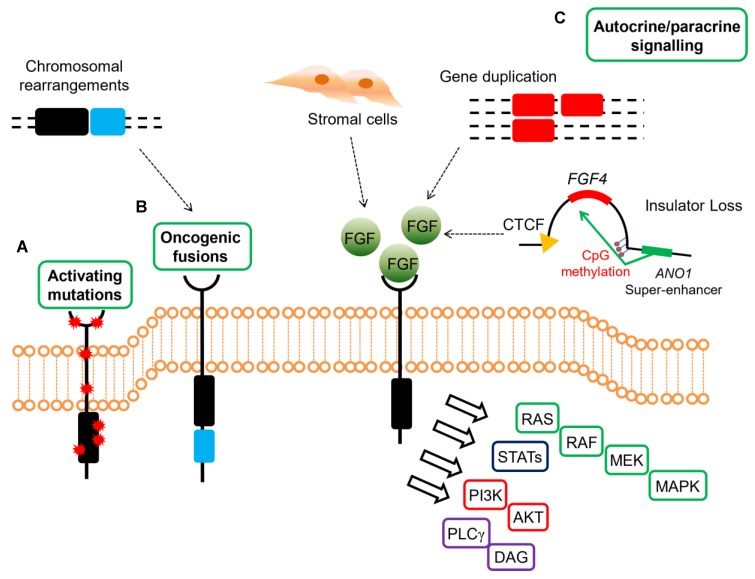
Mechanisms of oncogenic FGFR signaling in gastrointestinal stromal tumors (GISTs). FGFR signaling contributes to oncogenesis through several ligand-dependent and -independent mechanisms in GISTs, signaling through RAS/MAPK, PI3K/AKT, signal transducers and activator of transcription (STATs), phospholipase Cγ (PLCγ). (**A**) Activating mutations resulting in increased kinase activity in absence of ligand are present in *quadruple* WT GISTs. (**B**) Chromosomal translocations drive the fusion of parts of FGFR1 to other genes, thereby resulting in receptor hyperactivation in a ligand-independent manner. These events were identified in *quadruple* WT GISTs. (**C**) FGFRs can be stimulated by their ligands either in a paracrine fashion, through FGFs produced by stromal cells, or in an autocrine manner, when FGFs are produced directly from the tumor cells that also express FGFRs. In *quadruple* WT GISTs, FGF4 is overexpressed through genomic duplication of the FGF4 locus, while SDH-*deficient* GISTs overexpress FGF3/FGF4 by an epigenetic mechanism linked to hyper-methylation of the FGF4 insulator that disrupts CTCF binding, thus allowing the ANO1 super-enhancer to activate the FGF genes. In KIT-mutant GISTs, a role for autocrine or paracrine FGFR signaling has been proposed driving imatinib resistance.

Similarly, the active investigation on the oncogenic mechanism driven by SDH loss that is shared by different tumor types such as GISTs, paragangliomas and pheochromocytomas, led to the discovery of FGFR ligand overexpression and activation also in SDH-*deficient* GISTs. Conversely, the need to identify alternative mechanisms of imatinib resistance in KIT-mutant GISTs showed that the activation of FGF/FGFR signaling pathway and the crosstalk with KIT receptor could represent a route for tumor cells to acquire secondary resistance towards imatinib (Figure 2).

## 4. FGFR Mutations and Gene Fusions

Activating alterations in FGF receptors include point mutations and gene fusions. These two rare events were found only in some cases of the so-called qWT GISTs [17]. The presence of these undoubted oncogenic events in tumors lacking any other known molecular alteration strongly supports the driver role of FGFR mutations in a small percentage of qWT GISTs. In fact, in a comprehensive genomic study of a total of 14 qWT GIST patients performed by using whole-exome sequencing (WES) and gene expression analysis, a FGFR1 somatic missense mutation (c.1638C > A; p.N546K) was found in only one case, being the only oncogenic event identified [22].

Similarly Shi et al. analyzed 17 KIT/PDGFRA/SDH/RAS-pathway WT GISTs, finding one patient with an activating missense FGFR1 mutation (p.K656E) and two gene fusions: One case harboring FGFR1–HOOK3 (in-frame fusion of FGFR1 intron 17 and HOOK3 intron-4) and two patients carrying FGFR1–TACC1 (in-frame fusion of FGFR1 intron 17 and TACC1 intron-6) [21]. All these alterations are known to be deleterious, thus suggesting that FGFR1 mutations are likely drivers in qWT GISTs. In particular, K656E is a gain-of-function kinase domain mutation resulting in increased transforming potential, while N546K alters FGFR1 auto-phosphorylation, resulting in increased kinase activity [48,49].

Overall, the search for the oncogenic driver events responsible for this subset of diseases allowed the identification of five cases harboring FGFR1 alterations, two with activating missense mutations and three cases with oncogenic gene fusions (Table 1).

## 5. FGFR Ligand Overexpression

The search of the driver oncogenic event of qWT GISTs led to the very recent identification of the first shared alteration among multiple samples. This result was preceded by a very first identification of a specific gene expression profile shared by qWT GIST samples, with enrichment of Polycomb target genes (in particular of the classes of PRC2 targets and H3K27-bound genes) and of cell cycle progression and MAPK signaling, as exemplified by increased expression of SKP2, CDK6, NTRK2, and FGF4 [18]. It was only in 2019 that a high-throughput copy number analysis in eight qWT GISTs was performed, highlighting the presence of a recurrent focal gain of one copy in chromosome 11q13.3 cytoband overlapping the FGF3/FGF4 locus in six samples (Table 1) [25]. Interestingly, the other two without the duplication were carriers of the previously mentioned FGFR1 point mutation or NF1/MAX inactivation. The cryptic duplication, that was absent in KIT/PDGFRA-mutant and SDH-*deficient* GISTs, led to the marked overexpression of FGF4 with respect to the other two categories. SDH-*deficient* GISTs, even if negative for the duplication, showed enhanced FGF4 expression with respect to KIT/PDGFRA-mutant [25]. Given that all *quadruple* WT GISTs expressed both FGFR1 and FGFR2, and showed phosphorylated-AKT, the existence of an oncogenic autocrine loop supporting tumor growth is conceivable. Indeed, FGFR ligands are known to support autocrine or paracrine activation of FGF receptors in different tumor histotypes, including breast, pancreatic, prostate, gastric, colorectal, lung, bladder, and ovarian cancers, as well as multiple myeloma, glioblastoma, melanoma, and hepatocellular carcinoma [46,50,51,52,53,54].

Recently, the relevance of FGF-based autocrine loop emerged also in SDH-*deficient* GISTs. By means of sequencing of CTCF binding sites on bisulfite-treated DNA and ChIP sequencing specific for chromatin regions bound by CTCF, coupled to high-throughput chromosome conformation capture analyses, Flavahan et al. showed that genomic hypermethylation induced by SDH loss disrupts CTCF binding at the DNA regions located on the boundaries of the FGF3/FGF4 locus (Table 1). CTCF is an insulator protein able to bind the boundaries of topologically associated domains (TADs) in the human genome, that are regions where DNA sequences preferentially contact each other and whose genes are generally co-regulated. Hypermethylation prevents CTCF binding, thus disrupting the TAD and allowing the adjacent ANO1 super-enhancer to contact and activate the FGF genes [26]. As a matter of fact, ANO1 encodes the GIST clinical biomarker also known as DOG-1, and actually, ANO1 is highly expressed in all GIST subtypes. Therefore, the FGF3–FGF4 locus topology is profoundly altered in SDH-*deficient* GISTs, with CTCF insulator loss allowing aberrant expression of FGFR ligand genes. As a matter of fact, SDH-*deficient* GISTs show marked upregulation of FGF3 and FGF4, coupled to high FGFR1 expression and evidence of MAPK signature activation. Moreover, BGJ-398, a potent and selective inhibitor of FGFR1–4, completely suppressed tumor growth in a PDX model derived from an SDH-*deficient* GIST [26].

**Figure 3:**
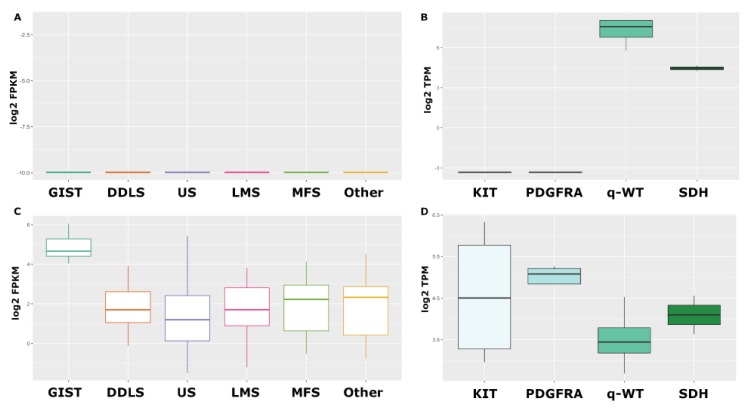
In silico analysis of FGFR ligands relevant in GIST pathogenesis. Expression of FGF4 in various subtypes of sarcomas (**A**) and in different molecular classes of GISTs (**B**). FGF2 expression in sarcomas (**C**) and in GIST samples (**D**). RNA sequencing data were taken from the CinSARC study on the GEO repository [55] and from Pantaleo et al. [22]. DDLS, Dedifferentiated Liposarcoma; US, Undifferentiated Sarcoma; LMS, Leiomyosarcoma; MFS, Myxofibrosarcoma. KIT, KIT-mutant GIST; PDGFRA, PDGFRA-mutant GIST; q-WT, *quadruple* WT; SDH, SDH-*deficient*. FPKM, Fragments Per Kilobase Million; TPM, Transcripts Per Kilobase Million.

Our results and those of Flavahan et al. indicate that FGF4 transcriptional activation is a shared event among SDH and qWT GISTs that could drive tumorigenesis. The very restricted expression of FGF4 in these specific and rare tumor settings can be further confirmed by exploiting public gene expression data. Using our own RNA sequencing data [22] together with those from the public repository derived from the CinSARC study [55], it is possible to demonstrate that FGF4 is not expressed in sarcomas (Figure 3A), which is somehow expected, since the expression of this growth factor is usually lost in human adult tissues (with the exception of testis). Likewise, FGF4 is not expressed in the largest part of GISTs (KIT/PDGFRA-mutant), except for SDH-*deficient* and qWT **(**Figure 3B). These data, taken together, highlight the relevance of FGF signaling in GISTs devoid of KIT/PDGFRA mutations or RAS-pathway activation, suggesting that FGF4 autocrine loop or FGFR mutations in SDH-*deficient* and qWT GISTs act as a surrogate of KIT/PDGFRA alterations in sustaining GIST tumor growth and spreading.

## 6. FGFR Events and Imatinib Resistance in KIT/PDGFRA Mutant GIST

Although imatinib has revolutionized the treatment of advanced GISTs, the majority of patients develop resistance to imatinib within 2 to 3 years. Even if acquired mutations in KIT or PDGFRA represent the most frequent event underlying secondary resistance, a signaling crosstalk between KIT and FGFR has been suggested as an alternative mechanism promoting resistance to imatinib [27]. This hypothesis was first proposed by Javidi-Sharifi and co-workers [27] who suggested that the development of resistance could be driven by the FGF2/FGFR3 loop. Interestingly, FGFR3 silencing restored imatinib response in imatinib-resistant GIST cells, whereas the addition of FGF2 to GIST cells induced KIT signaling, increased cell proliferation and desensitized GIST cells to imatinib, suggesting the existence of a crosstalk between the two receptors. Notably, the authors also found that rescue of cell growth by FGF2 was ineffective after KIT knockdown, further supporting the requirement of both KIT and FGFR3 presence for the induction of drug resistance. Additionally, FGF2-induced imatinib resistance remained effective after FGFR1 and FGFR2 silencing, whereas FGFR3 silencing ablated the response to FGF2, supporting the role of the FGF2/FGFR3 loop in mediating drug resistance. Conversely, siRNA-mediated knockdown of FGF2 re-sensitized GIST cells to imatinib, while increased protein levels of FGF2 were found in imatinib-resistant with respect to imatinib-sensitive GIST cells, a finding that was also confirmed in GIST patient samples [27]. Similarly, Li et al. have confirmed that FGF2 rescues the effect of KIT inhibition and reduces the sensitivity of imatinib in GIST cells, and conversely, the antiproliferative activity of imatinib was enhanced by cotreatment with BGJ398, a potent FGFR1–3 inhibitor, alone or in the presence of added FGF2 [28]. They have also shown that a prolonged exposure of KIT-mutant GIST cells to imatinib is associated with ERK signaling due to FGFR activation, and that the ERK rebound can be repressed by FGFR inhibition [28]. Altogether, these data show that FGFR-mediated reactivation of the MAPK signaling pathway attenuates the antiproliferative effects of imatinib in GISTs. The putative role of FGFR signaling through FGF2 in KIT-mutant GISTs can be supported also by gene expression data metanalysis, which suggest that GISTs express FGF2 at levels significantly higher than other sarcomas (Figure 3C) and that, among GISTs, KIT-mutant samples exhibit the largest heterogeneity of expression (Figure 3D).

The crosstalk between KIT and FGFR in playing an important role in imatinib resistance has been also extensively studied by Boichuck and co-workers [29,30,31,32]. In fact, they found that FGFR inhibition reduced cellular viability, induced apoptosis, and affected the growth kinetics of imatinib-resistant GIST cells in vitro. In contrast, imatinib-naive parental cells were not susceptible to FGFR inhibition, whereas inhibition of FGF-signaling restored the susceptibility to imatinib in resistant GIST cells [31]. Moreover, the authors tested the efficacy of BGJ398 in xenograft models of GISTs exhibiting secondary imatinib resistance due to loss of KIT/gain of FGFR2 [32]. Interestingly, they showed that inhibition of FGFR signaling by BGJ398 combined with imatinib treatment had a striking effect on tumor growth in vivo, suggesting that patients with advanced and metastatic resistant GISTs may benefit from therapeutic inhibition of FGFR signaling. Furthermore, the inhibition of FGF signaling pathway can sensitize GIST tumor cells to low doses of chemotherapeutic agents, such as topoisomerase II inhibitors [29]. In fact, the authors have proven for the first time that the inhibition of the FGF-signaling pathway in imatinib-resistant GISTs can interfere with the efficiency of DNA-damage-response signaling and attenuate the process of homology-mediated DNA repair, providing a rationale for GIST sensitization to DNA damaging agents [30].

## 7. Clinical Perspectives

Given that the deregulation of FGFR signaling has been observed in a subset of many cancers, in the last decade, several multi-target compounds that inhibit vascular endothelial growth factor and PDGF receptors, in addition to FGFRs, have been developed. Many such inhibitors, both multi-target as well as FGFR-specific tyrosine kinase inhibitors (TKIs), monoclonal antibodies, and FGF ligand traps are already in clinical trials or already approved for the treatment of many cancers (Table 2) [45,46].

Currently regorafenib, an oral multi-kinase inhibitor that blocks the activity of multiple protein kinases, including FGFR, is the only one approved in metastatic-resistant GISTs, after failure of imatinib and sunitinib [3]. Notably, it has been shown that, together with patients carrying the *KIT* exon 11 altogether with exon 17 activation loop resistance mutation, SDH-*deficient* GISTs experienced greater clinical benefit from regorafenib, suggesting that this subtype may be more addicted to FGF/FGFR signaling, besides its known indolent behavior [57]. According to this biological rationale, the ongoing REGISTRI trial is evaluating if KIT/PDGFRA WT may benefit from regorafenib upfront in first line, however the slow patients’ accrual due to the rarity of the studied population does not still allow to draw any definitive conclusion [56].

Beside regorafenib, in the last decade other multi-TKIs active also against FGFR, such as dovitinib, masitinib, ponatinib and pazopanib, have been tested in GISTs [58,59,60,61,62,63,64,65].

In a phase I study, a patient with imatinib and sorafenib-refractory GIST treated with dovitinib, a multi-kinase inhibitor targeting FGFR-1, FGFR-2 and FGFR-3, had a stable disease for eight months with a metabolic response by positron emission tomography (PET) scan during therapy, and a significant symptomatic improvement [58]. Subsequently, in a multicenter phase II trial (DOVIGIST) evaluating the anti-tumor activity of dovitinib as second-line treatment of patients with imatinib-resistant GIST, the objective response rate (ORR) was 5.3% and the median progression-free survival (PFS) was 4.6 months (90% CI, 2.8–7.4 months) [59]. Even if approximately 57% of patients who had a stable disease were KIT-mutant, the small sample size does not allow to draw conclusions about the efficacy of dovitinib in different mutational subsets.

Masitinib, another multi-TKI that inhibits also FGFR3, showed promising activity in a subset of patients with GISTs in a phase I clinical trial, and then in a phase II study on 30 patients with metastatic imatinib-naïve GISTs [60,61]. At 2 months, the response rate (RR) was 20%, with a median time-to-response of 5.6 months (0.8–23.8 months). The estimated median PFS was 41.3 months with a PFS rate of 59.7% and 55.4% at two and three years, respectively. The overall survival (OS) at 2 and 3 years was stable at 89.9% [61]. Masitinib has been also evaluated in patients with advanced GISTs after imatinib failure or intolerance, in a prospective, multicenter, randomized, open-label, two-parallel group, phase II study [62]. The study showed a positive benefit–risk ratio in favor of masitinib, thanks to the encouraging long-term median overall survival (OS) data and to its better safety profile, when compared against sunitinib [62].

Ponatinib, a multi-TKI targeting all FGFR members, has shown a broad inhibitory profile against KIT, with strong potency predicted against KIT with primary mutations in exon 11 coupled with most frequent secondary mutations, including those in the A-loop and the T670I gatekeeper residue [63]. Besides its potent activity against most major clinically relevant *KIT* mutations, several studies highlight ponatinib as a multi-targeted inhibitor, which acts as a pan-FGFR inhibitor in different FGFR-amplified or FGFR-mutated cancer cell lines [69]. This could broaden its clinical application also in other molecular subgroups of GISTs devoid of alternative lines of therapy.

Pazopanib, a multi-TKI targeting FGFR1, showed limited activity in heavily pretreated patients with advanced GISTs, refractory or intolerant to imatinib or sunitinib, in a multicenter phase II study, with a prolonged disease control after 17 cycles in only one patient with SDH-*deficient* GIST [64]. In a randomized, open-label phase 2 study (PAZOGIST), designed to assess the efficacy of pazopanib plus best supportive care versus best supportive care alone in resistant, unresectable, locally advanced, or metastatic GIST, pazopanib plus best supportive care showed an improvement of PFS at 4 months [65].

The activity of lenvatinib, another broad spectrum TKI targeting KIT, RET, PDGFRA, VEGFR 1–3 and FGFR 1–4 receptor tyrosine kinases, in still under evaluation in a multicenter, placebo-controlled, double-blinded phase II study of imatinib and sunitinib-refractory GIST patients (LENVAGIST) [66].

The recent accumulating evidence of the specific role of the FGF/FGFR pathway in almost all molecular subgroups of GIST patients has raised much more interest in the evaluation of selective FGFR inhibitors, besides the known multi-TKIs. However, the only available results came from a single-center, phase IB study, evaluating the combination of BGJ398, a selective FGFR1–4 inhibitor, with imatinib in patients with imatinib-refractory advanced GISTs [67]. Specifically, sixteen patients were enrolled in the protocol, which was closed prematurely due to toxicity of the drug combination. Clinical response was limited, with stable disease as the best response observed in seven patients by RECIST 1.1, and a median PFS of 12.1 weeks with the combination therapy, that is anyway superior to that achieved with imatinib re-challenge [67]. The study population included a series of heavily pre-treated KIT-mutant patients, irrespective of molecular-based selection, and the high toxicity encountered with the drug combination does not allow to draw conclusions on specific molecular subtypes’ response to FGFR inhibition. Indeed, it would be interesting to test the efficacy of this inhibitor in SDH-*deficient* or *quadruple* WT GISTs that showed molecular cues for addiction to FGF/FGFR signaling.

## 8. Conclusions

Taken together, all these findings show that the FGF/FGFR pathway activation may play a role in the pathogenesis of specific molecular subsets of GISTs, therefore its inhibition may have some windows of opportunity also in GIST management. However, the FGF/FGFR pathway inhibition in GISTs is still far from clinical practice, and necessarily requires stronger evidences for future development.

## Figures and Tables

**Table 1:** FGF/FGFR events in GIST.

	*n* Cases	FGF/FGFR Alteration	Methodology	GIST Classification
Shi et al., 2016 [21]	1	FGFR1 K656E	Target gene panel	*quadruple* WT GIST
	2	FGFR1-TACC1	Target gene panel	*quadruple* WT GIST
	1	FGFR1-HOOK3	Target gene panel	*quadruple* WT GIST
Pantaleo et al., 2017 [22]	1	FGFR1 N546K	Whole exome seq	*quadruple* WT GIST
Urbini et al., 2019 [25]	6	FGF4 duplication and overexpression	SNP-array, RNA sequencing	*quadruple* WT GIST
Flavahan et al., 2019 [26]	19 *	FGF insulator methylation and FGF3/FGF4 overexpression	CTCF ChIP and bisulfite seq, HiC and 4C, RNA sequencing	SDH-*deficient* GIST

* eight cases were analyzed by Chromatin Immunoprecipitation (ChIP) for CTCF (CCCTC-binding factor), bisulfite sequencing, and High-throughput Chromatin Conformation Capture (HiC), while eleven cases were subjected to locus-specific bisulfite sequencing of the FGF4 insulator.

**Table 2:** Current status of therapies targeting the FGF signaling pathway, describing the drug type, the target specificity, and the reference of the clinical trial on GIST.

Drug	Target	References in GIST
**Multi-kinase inhibitors**		
Regorafenib	FGFR1, VEGFR1,2.3 TIE2, KIT, RET, RAF-1, BRAF, and BRAF V600E, PDGFRβ	[3,56,57]
Dovitinib	FGFR1/3, VEGFR1/2/3, PDGFRβ, KIT, RET	[58,59]
Masitinib	FGFR3, PDGFRα/β, Lck, FAK	[60,61,62]
Ponatinib	FGFR1/2/3/4, Abl, PDGFRα/β, RET, KIT, CSF1R, FLT3, VEGFR1/2/3	[63]
Pazopanib	FGFR1, VEGFR1/2/3, PDGFRβ, KIT	[64,65]
Lenvatinib	FGFR1/2/3, VEGFR1/2/3, RET, KIT	[66]
Nindetanib	FGFR1, VEGFR2/3, PDGFRα	-
Lucitanib	FGFR1, VEGFR1/2, PDGFRα/β,Src	-
**FGFR inhibitors**		
Erdafitinib	FGFR1/2/3/4	-
Futibatinib	FGFR1/2/3/4	-
LY2874455	FGFR1/2/3/4	-
Rogaratinib	FGFR1/2/3/4	-
PRN1371	FGFR1/2/3/4	-
Infigratinib	FGFR1/2/3	[67]
Pemigatinib	FGFR1/2/3	-
Debio 1347	FGFR1/2/3	[68]
AZD4547	FGFR1/2/3	-
Roblitinib	FGFR4	-
H3B-6527	FGFR4	-
Fisogatinib	FGFR4	-
**Ligand trap and antibodies**		
FP-1039	FGF2	-
FPA114	FGFR2-IIb	-
MFGR1877S	FGFR3	-
